# *Staphylococcus aureus* nasopharyngeal carriage in rural and urban northern Vietnam

**DOI:** 10.1093/trstmh/tru132

**Published:** 2014-09-03

**Authors:** Kinh Van Nguyen, Tianying Zhang, Bich Ngoc Thi Vu, Trinh Tuyet Dao, Toan Khanh Tran, Diep Ngoc Thi Nguyen, Huong Kieu Thi Tran, Chuc Kim Thi Nguyen, Annette Fox, Peter Horby, Heiman Wertheim

**Affiliations:** a National Hospital for Tropical Diseases, Hanoi, Vietnam; b School of Clinical Medicine, University of Cambridge, Cambridge, UK; c Oxford University Clinical Research Unit, Hanoi, Vietnam; d Hanoi Medical University, Hanoi, Vietnam; e Nuffield Department of Clinical Medicine, Centre for Tropical Medicine, University of Oxford, Oxford, UK

**Keywords:** Carriage, MRSA, Nose, *Staphylococcus aureus*, Throat, Vietnam

## Abstract

**Background:**

*Staphylococcus aureus* is a common human pathogen that can colonise the respiratory tract and cause infection. Here we investigate the risk factors associated with nasopharyngeal carriage of *S. aureus* (including methicillin-resistant *S. aureus* [MRSA]) in Vietnam.

**Methods:**

Between February and June 2012, nasal and pharyngeal swabs for *S. aureus* culture, and demographic and socioeconomic data were taken from 1016 participants in urban and rural northern Vietnam, who were randomly selected from pre-specified age strata.

**Results:**

Overall *S. aureus* prevalence was 303/1016 (29.8%; adjusted for age: 33.8%). Carriage in the main cohort was found to be associated with younger age (≤5 years [OR 3.13, CI 1.62–6.03]; 6–12 [OR 6.87, CI 3.95–11.94]; 13–19 [OR 6.47, CI 3.56–11.74]; 20–29 [OR 4.73, CI 2.40–9.31]; 30–59 [OR 1.74, CI 1.04–2.92); with ≥60 as reference), living in an urban area (OR 1.36, CI 1.01–1.83) and antibiotics use (OR 0.69, CI 0.49–0.96). MRSA was detected in 80/1016 (7.9%). Being aged ≤5 years (OR 4.84, CI 1.47–15.97); 6–12 (OR 10.21, CI 3.54–29.50); 20–29 (OR 4.01, CI 1.09–14.77) and wealth (>3/5 wealth index, OR 1.63 CI 1.01–2.62) were significant risk factors for MRSA carriage.

**Conclusions:**

Nasopharyngeal carriage of *S. aureus* is present in one-third of the Vietnamese population, and is more prevalent among children. Pharyngeal carriage is more common than nasal carriage. Risk factors for *S. aureus* (including MRSA) carriage are identified in the community.

## Introduction

*Staphylococcus aureus* is an opportunistic pathogen that frequently colonises the human host, in particular the anterior nares and the skin.^1^ *S. aureus* has been associated with a wide range of pathologies including skin infections, septicaemia and respiratory infections.^2^ Methicillin-resistant *S. aureus* (MRSA) is an increasing problem in many developed countries, with a great amount of resources spent on its surveillance and prevention. These actions have had considerable success in the UK.^3^ *S. aureus* also causes significant mortality in resource limited regions of Asia, but there is limited information on its carriage and disease burden.^4,5^

The prevalent theory is that *S. aureus* colonisation increases the risk of *S. aureus* infection, and indeed carriage of *S. aureus* in the nose is linked to subsequent *S. aureus* infection.^6^ MRSA accounts for a significant proportion of *S. aureus* infections, estimated as high as 74.1% of all hospital acquired and 30.1% of all community acquired *S. aureus* infections in Vietnam.^7^ Intensive epidemiological studies have investigated *S. aureus* carriage in the population, though mostly in Western countries. Reported prevalence of *S. aureus* carriage in adults has varied widely in different populations.^1,8^ Discovering the epidemiology and risk factors of *S. aureus* and MRSA carriage is important, but has not yet been done comprehensively with all ages in Vietnam. Here we set out to investigate *S. aureus* and MRSA carriage rates in urban and rural Vietnam.

## Materials and methods

### Subjects

The study population consisted of children and adults enrolled in on-going demographic and health surveillance sites (DHSS) in Dong Da (urban) and Ba Vi (rural) districts of Hanoi. Potential participants were randomly selected from the cohort database and invited to participate by information leaflets and direct contact with study staff. Patients were enrolled when informed consent was obtained.

Dong Da district is an urban district of Hanoi and has a population of 352 000 people with a typical urban Vietnamese socioeconomic structure. Communes within Dong Da were classified according to high, low or middle income, and three communes were selected as representative of those three income levels, with 37 308 inhabitants living in 10 608 households in total. Ba Vi is a rural district with farming as the main occupation. The study site includes 50 000 people in nearly 13 000 households in 67 randomly selected clusters.

This carriage study was designed to collect nose and throat swabs, and demographic and health data in a representative sample of pre-specified age categories (approx. 100 individuals per category: ≤5 years, 6–12, 13–19, 20–29, 30–59 and ≥60 years) at each site, with a male: female ratio of 1. Exclusion criteria were insufficient sociodemographic data and unable to perform throat swab or nose swab. Within each age and gender category, subjects were randomly selected from the existing DHSS databases.

### Data collection

Using a standardised questionnaire, a variety of data postulated or known to influence *S. aureus* carriage were collected. These included: demographic data (age, sex), smoking status, presence of chronic diseases (diabetes, renal disease, liver disease, heart disease, malignancy, and lung diseases), recent (within the last 4 weeks) or current antibiotic use, occupation, education level, wealth information (housing and assets) and household size. Occupation was classified as within primary sector (farmer, hired labour), secondary sector (worker, handicraft), tertiary sector (government, services and trader), unemployed, student and unknown. Education was further classified as not enrolled in school (<6 years old, ≥6 years old and not enrolled in school), primary school or less (illiterate, literate but not a school graduate, ≤5 years in the new education system, ≤4 years in the old education system), middle school (6–9 years in the new education system, 5–7 years in old system), high school (10–12 years in new system, 8–10 years in old system) and higher education (college, university). Parents provided information for their children up to the age of 15 years.

### Microbiological methods

Nasal swabs of the anterior nares and throat swabs were collected using sterile Dacron swabs (Copan, Brescia, Italy). For the throat, the swab was dabbed firmly against the whole of posterior pharynx and tonsillar areas. Subsequently it was left in place for 5–10 seconds to absorb secretions. Both swabs were collected and transported on the same day to the microbiology laboratory of the National Hospital of Tropical Diseases. The swabs were plated on Phenol Mannitol agar which were incubated at 37°C for 48 hours and read on days 1 and 2. Suspect *S. aureus* isolates were identified via morphology of colony, Gram stain, coagulase and catalase testing. Methicillin resistance was determined by cefoxitin disk diffusion on Mueller Hinton agar plates according to the CLSI 2012 criteria^9^ and confirmed by real time PCR to identify the MecA gene as described elsewhere.^10^

### Statistical analysis

Univariate analysis was performed to identify risk factors for carriage using Pearson's χ^2^ test or Fisher's exact test when appropriate. Potential explanatory variables with p<0.1 in the univariate analysis were included in a logistic regression model, followed by stepwise variable selection. Odds ratio (OR) and 95% CIs were calculated using univariate analysis and multivariate analysis adjusted for age. Multiple comparisons of percentages were adjusted using the Bonferroni correction. p<0.05 was considered as significant (two sided). All calculations were performed using R package (R-project, Vienna, Austria; version 3.0.1). Percentage prevalence for the sample population was adjusted for Vietnamese age structure data provided in the 2009 Vietnam Population and Housing Census.^11^ Wealth status was quantified using a wealth index score (quintiles–1 is the poorest, 5 is the wealthiest) estimated by principle component analysis. It uses a set of correlated variables describing housing conditions and household ownership assets.

## Results

Data and samples from 1029 participants were collected between February and June 2012. We did not have any age data for 13 participants and they were excluded from the cohort (n=1016). Characteristics of the cohort are shown in Table 1. There were 354 participants aged <20 years old (34.8%) and 662 participants aged ≥20 years (65.2%); 38.8% (394) of participants were from the urban area of Dong Da while the remainder were from the rural area of Ba Vi; 54.0% (549) of participants were male and 46.0% (467) were female. Overall carriage rate for *S. aureus* was 29.8% (CI 27.0–32.8) or 33.8% (CI 29.4–38.8) adjusted for age, representing 303 positive participants (Table 2). For participants aged ≥20 years, prevalence was 28.8% (CI 23.3–35.4) adjusted for age structure. For those aged under 20 year old, the adjusted prevalence was 43.1% (CI 36.2–50.9).

**Table 1.** Study participants' age, gender and living location

Age (years)	Gender		Living location		Total, n=1016 n (%)
	Male, n=549 n (%)	Female, n=467 n (%)	Dong Da, n=394 n (%)	Ba Vi, n=622 n (%)	
≤5	40 (47.1)	45 (52.9)	26 (30.6)	59 (69.4)	85 (8.4)
6–12	91 (55.2)	74 (44.8)	66 (40.0)	99 (60.0)	165 (16.2)
13–19	44 (42.3)	60 (57.7)	58 (55.8)	46 (44.2)	104 (10.2)
20–29	26 (41.3)	37 (58.7)	29 (46.0)	34 (54.0)	63 (6.2)
30–59	275 (61.8)	170 (38.2)	126 (28.3)	319 (71.7)	445 (43.8)
≥60	73 (47.4)	81 (52.6)	89 (57.8)	65 (42.2)	154 (15.2)

**Table 2.** Characteristics of *Staphylococcus aureus* (MSSA and MRSA) carriers vs non-carriers (n=1016)

Risk factor	Both MSSA and MRSA positive, n=303	SA negative, n=713	MRSA positive, n=80	MSSA positive, n=223	OR (95% CI) SA carriage vs non-carriage	OR (95% CI) MRSA carriage vs MSSA carriage and non-carriage
	n (%)	n (%)	n (%)	n (%)		
Age (years)						
≤5 (n=85)	26 (30.6)	59 (69.4)	10 (11.8)	16 (18.8)	2.64 (1.39–5.04)	5.0 (1.52–16.5)
6–12 (n=165)	84 (50.9)	81 (49.1)	37 (22.4)	47 (28.5)	6.22 (3.61–10.73)	10.8 (3.8–31.2)
13–19 (n=104)	53 (51.0)	51 (49.0)	5 (4.8)	48 (46.2)	6.24 (3.45–11.28)	1.90 (0.50–7.23)
20–29 (n=63)	27 (42.9)	36 (57.1)	6 (9.5)	21 (33.3)	4.50 (2.30–8.82)	3.95 (1.07–14.5)
30–59^a^ (n=445)	91 (20.4)	354 (79.6)	18 (4.0)	73 (16.4)	1.54 (0.93–2.56)	1.58 (0.53–4.75)
≥60 (n=154)	22 (14.3)	132 (85.7)	4 (2.6)	18 (11.7)	REF	REF
Living location						
Dong Da (urban) (n=394)	137 (34.8)	257 (65.2)	27 (6.9)	110 (27.9)	1.46 (1.10–1.94)	0.79 (0.47–1.31)
Ba Vi (rural) (n=622)	166 (26.7)	456 (73.3)	53 (8.5)	113 (18.2)	REF	REF
Gender						
Male (n=549)	164 (29.9)	385 (70.1)	39 (7.1)	125 (22.8)	1.00 (0.76–1.33)	0.79 (0.49–1.29)
Female (n=467)	139 (29.8)	328 (70.2)	41 (8.8)	98 (21.0)	REF	REF
Chronic diseases						
Present (n=216)	54 (25.0)	162 (75.0)	13 (6.0)	41 (19.0)	0.74 (0.51–1.05)	0.70 (0.35–1.31)
Not present (n=800)	249 (31.1)	551 (68.9)	67 (8.4)	182 (22.8)	REF	REF
Smoking						
Smoker (n=172)	36 (20.9)	136 (79.1)	5 (2.9)	31 (18.0)	0.58 (0.38–0.87)	0.31 (0.097–0.78)
Non smoker (n=836)	263 (31.5)	573 (68.5)	73 (8.7)	190 (22.7)	REF	REF
Unknown	4	4	2	2	NA	NA
Recent/current antibiotic use						
Positive (n=277)	68 (24.5)	209 (75.5)	25 (9.0)	43 (15.5)	0.69 (0.50–0.96)	1.20 (0.70–2.01)
Negative (n=721)	230 (31.9)	491 (68.1)	55 (7.6)	175 (24.3)	REF	REF
Unknown	5	13	0	5	NA	NA
Ethnicity						
Kinh (n=607)	162 (26.7)	445 (73.3)	52 (8.6)	110 (18.1)	0.69 (0.52–0.92)	1.27 (0.77–2.14)
Muong (n=16)	3 (18.8)	13 (81.3)	0	3 (18.8)	0.54 (0.10–1.98)	NA
Dao	1	0	1	0	NA	NA
Other (n=392)	137 (34.9)	255 (65.1)	27 (6.9)	110 (28.1)	REF	REF
Occupation^b^						
Primary sector (n=214)	45 (21.0)	169 (79.0)	11 (5.1)	34 (15.9)	0.56 (0.38–0.81)	0.58 (0.27–1.12)
Secondary sector (n=17)	4 (23.5)	13 (76.5)	0	4 (23.5)	0.72 (0.17–2.36)	NA
Tertiary sector (n=150)	39 (26.0)	111 (74.0)	10 (6.7)	29 (19.3)	0.80 (0.53–1.20)	0.81 (0.36–1.63)
Student (n=250)	131 (52.4)	119 (47.6)	32 (12.8)	99 (39.6)	3.80 (2.78–5.19)	2.19 (1.32–3.60)
Below school age (n=118)	44 (37.3)	74 (62.7)	21 (17.7)	23 (19.5)	1.47 (0.96–2.22)	3.07 (1.70–5.4)
Retired (n=102)	18 (17.6)	84 (82.4)	2 (2.0)	16 (15.7)	0.47 (0.26–0.81)	0.21 (0.025–0.82)
Others (n=165)	22 (13.3)	143 (86.7)	4 (2.4)	18 (10.9)	ND	ND
Household size						
Large (>4) (n=431)	129 (29.9)	302 (70.1)	38 (8.8)	91 (21.1)	1.01 (0.76–1.34)	1.25 (0.77–2.03)
Small (n=585)	174 (29.7)	411 (70.3)	42 (7.2)	132 (22.6)	REF	REF
Education^b^						
Not enrolled in school (n=121)	44 (36.4)	77 (63.6)	21 (17.4)	23 (19.0)	1.40 (0.92–2.12)	2.97 (1.64–5.21)
Primary school or less (n=216)	85 (39.4)	131 (60.6)	29 (13.4)	56 (25.9)	1.73 (1.25–2.40)	2.28 (1.35–3.77)
Middle school (n=347)	86 (24.8)	261 (75.2)	13 (3.7)	73 (21.0)	0.69 (0.51–0.93)	0.35 (0.17–0.65)
High school (n=187)	52 (27.8)	135 (72.2)	11 (5.9)	41 (21.9)	0.89 (0.61–1.28)	0.69 (0.32–1.35)
Higher education (n=145)	36 (24.8)	109 (75.2)	6 (4.1)	30 (20.7)	0.75 (0.48–1.13)	0.46 (0.20–1.09)
Wealth (Wealth Index: 1=Poorest, 5=Richest)						
Rich (>3) (n=427)	136 (31.9)	291 (68.1)	43 (10.1)	93 (21.8)	1.18 (0.89–1.56)	1.71 (1.05–2.80)
Poor (n=586)	166 (28.3)	420 (71.7)	36 (6.1)	130 (22.2)	REF	REF
Unknown	1	2	1	0	NA	NA

MRSA: methicillin-resistant *S. aureus*; MSSA: methicillin-susceptible *S. aureus*; ND: not done; NS: Not significant (p>0.05); REF: Reference; SA: *Staphylococcus aureus*.

^a^ More patients were recruited in this age group as longer time span.

^b^ Reference for occupation and education are the rest of the population.

### Carriage site

We swabbed nose and throats separately and categorised positive carrier swabs as follows: nose only carrier, throat only carrier, or nose and throat carrier. Prevalence of nose only carriers, throat only carriers, and nose and throat carriers were 88/1016 (8.7%, CI 7.0–10.6), 141/1016 (13.9%, CI 11.8–16.2) and 73/1016 (7.2%, CI 5.7–9.0) respectively. Percentage *S. aureus* positive rate in different swab categories are shown in Table 3. Overall, throat only colonisation had the highest prevalence in all of the age groups apart from those ≥60 years. The prevalence of colonisation of both nose and throat was highest amongst the population aged between 6–12 years (20.0%, CI 14.3–27.1). The highest prevalence of throat only *S. aureus* colonisation was in the 13–19 years (35.6%, CI 26.6–45.6) and 20–29 years (31.7%, CI 20.9–44.8) age groups. In the ≥30 year olds there were no significant differences in percentage positive rate in all three categories of swabs. In the Dong Da urban population, having throat only *S. aureus* positive swabs was the most prevalent (19.5%, CI 15.8–23.9; p<0.001 compared to all other swab categories). In Ba Vi there were no significant differences between the swab categories.

**Table 3.** Significant risk factors of study participants with positive carriage by swab categories^a^ (n=1016)

Risk factor	Nose swab positive only (n=88, 8.7%, 95% CI 7.0–10.6)	Throat swab positive only (n=141, 13.9%, 95% CI 11.8–16.2)	Both swabs positive (n=73, 7.2%, 95% CI 5.7–9.0)
	n (%, CI)	n (%, CI)	n (%, CI)
Age (years)			
≤5 (n=85)	9 (10.6, 5.3–19.6)	10 (11.8, 6.1–21.0)	7 (8.2, 3.7–16.8)
6–12 (n=165)	21 (12.7, 8.2–19.0)	30 (18.2, 12.8–25.1)	33 (20.0, 14.3–27.1)
13–19 (n=104)	7 (6.7, 3.0–13.6)	37 (35.6, 26.6–45.6)	9 (8.7, 4.3–16.2)
20–29 (n=63)	4 (6.3, 2.1–16.3)	20 (31.7, 20.9–44.8)	3 (4.8, 1.2–14.2)
30–59 (n=445)	36 (8.1, 5.8–11.1)	37 (8.3, 6.0–11.4)	17 (3.8, 2.3–6.2)
≥60 (n=154)	11 (7.1, 3.8–12.7)	7 (4.5, 2.0–9.5)	4 (2.6, 0.8–6.9)
Living location			
Dong Da (urban) (n=394)	27 (6.9, 4.6–9.9)	77 (19.5, 15.8–23.9)	33 (8.4, 5.9–11.7)
Ba Vi (rural) (n=622)	61 (15.5, 12.1–19.5)	64 (16.2, 12.8–20.3)	40 (10.2, 7.4–13.7)
Gender			
Male (n=549)	43 (7.8, 5.8–10.5)	84 (15.3, 12.4–18.6)	36 (6.6, 4.7–9.0)
Female (n=467)	45 (9.6, 7.2–12.8)	57 (12.2, 9.4–15.6)	37 (7.9, 5.7–10.9)
Chronic diseases			
Present (n=216)	26 (12.0 , 8.2–17.3)	16 (7.4, 4.4–12.0)	11 (5.1, 2.7–9.2)
Not present (n=800)	62 (7.8, 6.0–9.9)	125 (15.6, 13.2–18.4)	62 (7.8, 6.0–9.9)
Smoking^b^			
Smoker (n=172)	13 (7.6, 4.3–12.8)	15 (8.7, 5.1–14.2)	8 (4.7, 2.2–9.3)
Non smoker (n=836)	72 (8.6, 6.8–10.8)	125 (15.0, 12.6–17.6)	65 (7.8, 6.1–10.0)
Recent/current antibiotic use^c^			
Positive (n=277)	21 (7.6, 4.9–11.5)	31 (11.2, 7.8–15.7)	16 (5.7, 3.4–9.4)
Negative (n=721)	64 (8.8, 7.0–11.3)	108 (15.0, 12.5–17.8)	57 (7.9, 6.1–10.2)

^a^ Swab type was not recorded for one *S. aureus* positive participant.

^b^ Smoking status unknown for 3 nose swab positive only and 1 throat swab positive only participants.

^c^ Antibiotic status unknown for 3 nose swab positive only and 2 throat swab positive only participants.

Non smokers had significantly more throat only positive participants (15.0%, 12.6–17.6, p<0.001) than the other categories. This is unlike the smokers, where there were no significant differences between the categories

### General determinants of *S. aureus* carriage

Risk factors for carriage of *S. aureus* are summarised in Table 2. Significant factors associated with *S. aureus* carriage were identified using univariate analysis: age, living in Dong Da, being a smoker, recent/current antibiotics use, Kinh ethnicity, primary sector occupation, being a student, education standard of primary school or less and middle school education. Being a student (occupation) was considered a strong confounding variable due to its age specific distribution. Indeed, ANOVA analysis shows that when corrected for the effect of age, being a student is not a significant explanatory variable (p=0.072). Hence to remove its masking effect on age, we excluded it in the multivariate analysis. Stepwise backward regression was performed with age kept in categories, and with the ≥60 years range as the reference. We found that younger age (≤5 years [OR 3.13, CI 1.62–6.03], 6–12 years [OR 6.87, CI 3.95–11.94], 13–19 years [OR 6.47, CI 3.56–11.74], 20–29 years [OR 4.73, CI 2.40–9.31], 30–59 years [OR 1.74, CI 1.04–2.92]), living in urban area Dong Da (OR 1.36, OR 1.01–1.83) and having current/recent antibiotics use (OR 0.69, CI 0.49–0.96) were independently explanatory variables for *S. aureus* carriage (Table 4).

**Table 4.** Multivariate analysis of significant risk factors for colonisation by *Staphylococcus aureus*

Risk factors	Total S*taphylococcus aureus* carriage (MSSA and MRSA combined; n=1016)			MRSA (n=1016)		
	OR	95% CI	p	OR	95% CI	p
Age (years) Reference range: ≥60 years						
≤5	3.13	1.62–6.03	<0.001	4.84	1.47–15.97	0.010
6–12	6.87	3.95–11.94	<0.001	10.21	3.54–29.50	<0.001
13–19	6.47	3.56–11.74	<0.001	1.72	0.45–6.60	NS
20–29	4.73	2.40–9.31	<0.001	4.01	1.09–14.77	0.037
30–59	1.74	1.04–2.92	0.035	1.50	0.50–4.50	NS
Living in Dong Da	1.36	1.01–1.83	0.043	NA	NA	NA
Current/recent antibiotic use	0.69	0.49–0.96	0.029	NA	NA	NA
Rich (>3/5 Wealth Index)	NA	NA	NA	1.63	1.01–2.62	0.046

NA: not applicable; NS: not significant (p>0.05).

**Figure 1.**
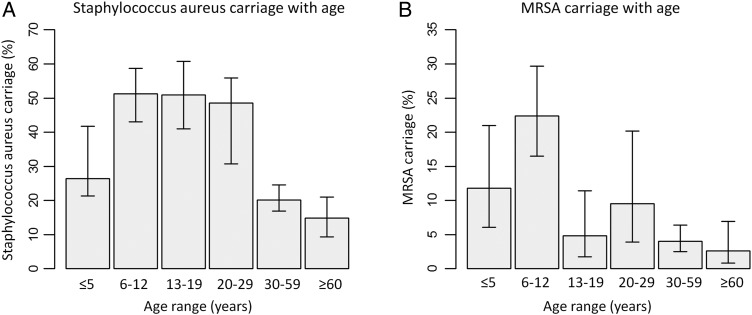
(A) *Staphylococcus aureus* carriage distribution with age. (B) MRSA carriage distribution with age. Error bars represent 95% CIs.

With age, there was a clear higher prevalence of *S. aureus* carriage between the ages of 6 and 29 years. The prevalence decreased after 29 years of age (Figure 1A). Participants below 5 years of age showed a lower carriage rate of *S. aureus* than expected (31%), as shown in Table 2.

### Antibiotic use

Antibiotics can influence bacterial carriage rate depending on their specific antibacterial activity. Participants aged ≤5 years had the highest percentage recent/current antibiotic use (41.2%; CI 30.8–52.4) among the age groups. Ba Vi participants had higher antibiotics use (31.5%; CI 27.9–35.3) than Dong Da participants (20.6%; CI 16.7–25.0). There was also significantly higher antibiotic use in those with chronic diseases (34.7%; CI 28.5–41.5) than those without (25.3%; CI 22.3–28.4).

With recent or current antibiotic use, prevalence differences between the three swab categories were not significant. However in populations without antibiotics, throat only carriage was significantly more prevalent than other swab categories (p=0.001 [nose only]). There was no record of which antibiotics were used in 179/277 (64.6%) antibiotic using participants. Out of the ones we have data for the following antibiotics were used: amoxicillin (n=33), cephalexin (17) and ampicillin (35), and none used fluoroquinolones.

### Methicillin-resistant *S. aureus* carriage

Overall MRSA carriage in the whole population was 7.9% (80/1016, CI 5.9–10.4) adjusted for age structure; 80/303 (26.4%; CI 21.6–31.8) of *S. aureus* identified were MecA positive. Those ≥20 years old had an adjusted prevalence of 5.4% (CI 3.3–8.8) while <20 year old had a higher prevalence of 12.3% (CI 9.0–16.5) when adjusted for age structure. Figure 1B shows the prevalence of MRSA in each age group. There was a trend of increased prevalence in childhood peaking at 6–12 years old, and significantly decreased prevalence in 13–19, 30–39 and ≥60 year age groups compared to the 6–12 years group.

Identified significant factors (Table 2) associated with MRSA carriage were age (p<0.001), smoking (p=0.0072), being a student (p<0.001), being below school age (p<0.001), retired (p= 0.018), not enrolled in school (p<0.001), primary school education or less (p=0.001), middle school education (p<0.001) and wealth index >3 (p=0.02). Again multivariate analysis was carried out with age kept in categories and ≥60 years as the reference. After multiple stepwise regression being aged ≤5 years (OR 4.84, CI 1.47–15.97); 6–12 years (OR 10.21, CI 3.54–29.50); 20–29 years (OR 4.01, CI 1.09–14.77) and having a wealth index of >3 (OR 1.63, CI 1.01–2.62) were significant risk factors (Table 4).

## Discussion

This is the first comprehensive community based study of *S. aureus* nasal and throat carriage in a large Asian population with all age categories represented. General prevalence was found to be 33.8% overall (adjusted for age), which was higher than in other population studies of mixed ages in Malaysia,^12^ USA,^8^ France, Moldova, Algeria and Cambodia.^13^ Prevalence in those aged ≥20 years was 28.8% adjusted for age, which is lower than in the USA^14^ and Malaysia.^15^ However it is higher than in some European countries including Austria, Belgium, Croatia, France, Hungary and Spain.^16^ For those <20 year old, the prevalence was 43.1% adjusted for age, which is higher than other estimates for children of approx. 35% in USA.^14^ The higher prevalence in our populations can be explained by the fact that most other studies used nasal swabs only to identify carriers while we used both nasal and throat swabs. Considering in our study 13.9% of carriers were throat swab positive only, our prevalence figures should indeed be higher than others.

As in other studies, age was a significant determinant of *S. aureus* carriage prevalence. Carriage peaked between 6–12 years and decreased in older and younger ages. This decreasing trend with age has been found in other papers.^1^ However we found high prevalence of *S. aureus* carriage even in the 20–29 years age range, suggesting that factors contributing to changes in carriage persevere well into early adulthood in Vietnam. Age range ≤5 years had a lower than expected prevalence rate. This can be explained by the fact that age range ≤5 years had the highest antibiotics usage rate for all age groups (41.2%). Young children are known to use a lot of antibiotics in Vietnam, with up to 62% of children in a community receiving antibiotics in a one-month period for mostly mild acute respiratory infections.^17^ A limitation is that the 20–29 year age category is underrepresented; due to work and family obligations this category was hard to recruit.

Other risk factors found for general population included living in an urban area (Dong Da). This is in contrast with other papers that have found living or working in a farm (rural area) was a risk factors for carriage.^18^ The difference within the main cohort analysis might be due to the higher antibiotic use in Ba Vi rural area (31.5% versus 20.6%). Having recent or current antibiotic use was a significant protective factor against carriage. The association of recent or current antibiotic therapy with decreased *S. aureus* carriage is not surprising as oral antibiotics may remove colonising commensals. Our data also showed that 27.3% of our participants had recent (within the last 4 weeks) or current antibiotic use. This presents a worrying picture as antibiotic use is the main driver behind resistance.^19^ We did not have detailed data on the kind and dose of antibiotics used and could therefore not do a more elaborate analysis. Fluorquinolones use was not reported. These antibiotics have been associated with risk of MRSA carriage.^20,21^

We found there was a distinctive pattern in *S. aureus* swab positive categories with age. Those aged 6–12 years old had the highest both nose and throat positive rate for *S. aureus* out of all age ranges (20.0%). It may suggest the younger age group may not only have higher carriage rates, but also greater number of sites colonised. Smokers had non-significant differences between *S. aureus* carriage categories positive rates compared to non-smokers, who had significantly more throat only colonisation (15.0%). This can be explained by smoking having a protective effect on *S. aureus* carriage, which was found in some other studies.^22^ Indeed, smoking was found to be significant in univariate analysis though in multivariate it was not, possibly due to the masking effect of interactions between smoking and other risk factors.

Our data did not demonstrate wealth or household size as significant factors that influence *S. aureus* carriage, unlike other papers.^23^ For household size, only 3.7% of participants had large households (>7), which may have influenced the association. Surprisingly, wealth was not found associated with general *S. aureus* carriage, but was significant for MRSA carriage. Other papers have shown that diabetes was associated with carriage.^1,24^ Unfortunately, we did not have enough diabetic patients (2.0%) in our cohort to be able to test such association. Furthermore, males did not have a higher *S. aureus* carriage rate in our participants in contrast to other papers.^18^

MRSA prevalence was 7.9% overall when adjusted for Vietnam age structure. The ≥20 year olds had an adjusted prevalence of 5.4%. This is similar to the prevalence found in adults in Taiwan^25^ and China.^26^ Those aged <20 years had an adjusted prevalence of 12.3%. This is much larger than reported childhood carriage rates reported in Cambodia.^27^ The prevalence for ≤5 year old children was 11.8%, which is slightly higher than found in studies on pre-school children in South Korea^28^ and Taiwan.^29^

Age was also a significant factor for MRSA carriage in the multivariable analysis. Prevalence was higher in the young, peaking with the age range of 6–12 years (22.4%). Prevalence decreased with age, significantly after 30 years of age compared to the 6–12 years age range. Interestingly, having a wealth index of >3 was a significant risk factor for MRSA colonisation. One might suspect this was because the wealthy have more access to health care professionals and settings, which are identified risk factors for MRSA colonisation.^30^ Unlike general *S. aureus* carriage, antibiotic use was found not to be a significant explanatory variable in MRSA carriage. This is perhaps not surprising as MRSA is resistant to a wide selection of antibiotics. A limitation is that we do not know the healthcare exposures of the screened participants.

A larger cohort is needed to explore some other associations of carriage seen in previous papers, such as diabetes. We were able to identify some risk factors for MRSA carriage despite the limitations of our sample size. However further studies are needed to determine whether carriage positive participants were intermittent or chronic carriers, as this might influence the risk of *S. aureus* infection.

### Conclusions

*S. aureus* nasopharyngeal carriage is present in roughly one-third (with approximately 25% MRSA) of the northern Vietnamese population, and is more prevalent among children. Pharyngeal carriage is more common than nasal carriage. Risk factors for carriage have been identified in the community, both for MSSA and MRSA.
